# Supplementary material for: Bidirectional histone monoaminylation dynamics regulate neural rhythmicity
Source: Nature. 2025 Jan 8;637(8047):974–82. doi: 10.1038/s41586-024-08371-3 (PMC11754111; doi:10.1038/s41586-024-08371-3)
Supplement: Supplementary file 2 — Reporting Summary [file 41586_2024_8371_MOESM2_ESM.pdf]

nature portfolio

Corresponding author(s): Ian Maze, PhD

Last updated by author(s): 11/01/2024

Reporting Summary

Nature Portfolio wishes to improve the reproducibility of the work that we publish. This form provides structure for consistency and transparency in reporting. For further information on Nature Portfolio policies, see our Editorial Policies and the Editorial Policy Checklist.

Statistics

For all statistical analyses, confirm that the following items are present in the figure legend, table legend, main text, or Methods section.

n/a	Confirmed	
☐	☒	The exact sample size (*n*) for each experimental group/condition, given as a discrete number and unit of measurement
☐	☒	A statement on whether measurements were taken from distinct samples or whether the same sample was measured repeatedly
☐	☒	The statistical test(s) used AND whether they are one- or two-sided<br>*Only common tests should be described solely by name; describe more complex techniques in the Methods section.*
☒	☐	A description of all covariates tested
☐	☒	A description of any assumptions or corrections, such as tests of normality and adjustment for multiple comparisons
☐	☒	A full description of the statistical parameters including central tendency (e.g. means) or other basic estimates (e.g. regression coefficient) AND variation (e.g. standard deviation) or associated estimates of uncertainty (e.g. confidence intervals)
☐	☒	For null hypothesis testing, the test statistic (e.g. *F*, *t*, *r*) with confidence intervals, effect sizes, degrees of freedom and *P* value noted<br>*Give P values as exact values whenever suitable.*
☒	☐	For Bayesian analysis, information on the choice of priors and Markov chain Monte Carlo settings
☒	☐	For hierarchical and complex designs, identification of the appropriate level for tests and full reporting of outcomes
☒	☐	Estimates of effect sizes (e.g. Cohen's *d*, Pearson's *r*), indicating how they were calculated

Our web collection on statistics for biologists contains articles on many of the points above.

Software and code

Policy information about availability of computer code

Data collection

No custom algorithms were used.

Data analysis

RNA-seq data analysis
Raw sequencing reads were demultiplexed using bcl2fastq2 (Illumina, v2.20). Samples were aligned to the GRCm38 (UCSC build GCA_000001635.2) mouse genome using STAR (v2.7.11b) alignReads in mode BAM SortedByCoordinate. Gene counts were generated using htseq-count (HTSeq v2.0.5) with the following parameters: --format=bam --minaqual=10 --type=exon --idattr=gene_name --stranded=yes --mode=union using the Ensembl v93 annotation. Gene counts were normalized using DEseq2 (v1.44.0) prior to analysis using JTKcycle (v3.1) to identify cycling genes from the dataset using the parameters jtkdist (varying depending on replicates), periods(2:6) and jtk.init(periods,4). Genes with an ADJ.P < 0.05 were deemed significant and Z-scores were computed in R using tidyverse (v2.0.0). heatmap.2 in R was used to visualize the cycle genes (gplots version 3.1.3.1) across the zeitgeber. Cyclic genes were further assessed using ChEA in Enrichr (https://maayanlab.cloud/Enrichr/, v3.2), which infers transcription factor regulation from integration of previous genome-wide chromatin immunoprecipitation (ChIP) analyses. Further ontology was also conducted using Enrichr (v3.2). Odds ratios were calculated using the GeneOverlap R package (Version 1.26.0). DEseq2 (v1.44.0) was run to perform pairwise differential expression analyses between H3.3 and GFP viral treated samples (to ensure limited to no significant differences between control groups; number of DEGs between GFP vs. H3.3 WT at: ZT0 = 1, ZT4 = 0, ZT8 = 0, ZT12 = 40, ZT16 = 1, ZT20 = 1) before combining them together to run JTKcycle, as previously described. Differentially expressed (DE) genes were defined at FDR < .01. Overlap of JTK cycle genes with peak lists from CUT&RUN-seq (see below) was performed in R using dplyr (v1.1.4). Individual circadian rhythm controlling genes identified in Enrichr (v3.2) were highlighted on heatmaps manually.

CUT&RUN-seq data analysis
Raw sequencing files were demultiplexed using bcl2fastq2 (Illumina, v2.20). Between 20-100 million total reads were achieved for each

replicate (average 42.6 million). Samples were aligned to the hg19 or mm10 genome using bowtie2 (2.5.0), with the following parameters: --local --very-sensitive-local --phred33 -I 10 -X 700 --dovetail --no-unal --no-mixed --no-discordant. Low quality reads were filtered out using Samtools (v1.9) with a cutoff MAPQ score of 30, and only unique reads were kept for further processing. Unique read files for each replicate/timepoint/antibody were merged and used for peak calling using MACS2 (v3.0.0a6) with the callpeaks function and the options -f BAMPE -q 0.05 --broad --broad-cutoff .05, using the corresponding IgG sample as the -c. For visualization, each sample was normalized by scaling the samples based off the E. coli spike-in DNA. Each sample was aligned to the E. coli genome (MG1655), and the uniquely aligned reads counted. The number of E. coli reads for each replicate between timepoints was compared, with the sample with the lowest number of E. coli reads set at a scaling factor of 1X. The other samples were scaled down by a scaling factor that was computed by dividing the lowest number of E. coli reads by the sample number of E. coli reads. This was done separately for each antibody, as an internal normalization between timepoints across zeitgeber time. The same was done comparing vehicle and zolpidem treated animals. Genome coverage tracks (bigwig files) were produced using deepTools (3.5.1) bamCoverage function with the options –binSize 10 –smoothLength 30 --normalizeUsing None --scaleFactor #(derived from E. coli spike in) and using an ENCODE hg19 or mm10 blacklist file (https://doi.org/10.1038/s41598-019-45839-z, hg19.v2/mm10v2) to discard regions with consistently non-specific signal. Peak annotation and motif analysis of MACS2 called peaks were performed using HOMER (v4.11). Heatmaps were made using Deeptools (v3.5.5) computeMatrix and plotHeatmaps in reference-point mode, centered over TSSs or peak centers, using binSize 10 and –sortUsing mean, sorted in descending order. TSSs were downloaded from the UCSC (mm10;gencode VM23/hg19;gencode V45lift37) table browser using the canonically annotated transcript for each gene. Overlap of various peaks and TSSs was achieved using bedtools intersect (v2.31). For generation of average plot profiles (+/-500 bp of TSS) and input for running JTK cycle on H3K4me3Q5his and H3K4me3Q5ser marks, Deeptools computeMatrix was run centered over TSSs with the parameters -a 500 -b 500 -binSize 100. The resulting matrix and coordinates files were merged in R using dplyr (v1.1.4), and the average signal over the 1kb window for each timepoint was computed for the plot profiles. For JTK cycle, the average signal over the same 1kb window was computed for all individual biological replicates, and JTK cycle (v3.1) was run with the options jtkdist(6,3), periods(2:6), and jtk.init(periods,4). Genes with a ADJ.P < 0.05 were further analyzed, with Z-scores being computed in R using tidyverse (v2.0.0) and plotted as a heatmap using the function heatmap.2 (gplots version 3.1.3.1).

For manuscripts utilizing custom algorithms or software that are central to the research but not yet described in published literature, software must be made available to editors and reviewers. We strongly encourage code deposition in a community repository (e.g. GitHub). See the Nature Portfolio guidelines for submitting code & software for further information.

Data

Policy information about availability of data

All manuscripts must include a data availability statement. This statement should provide the following information, where applicable:

- Accession codes, unique identifiers, or web links for publicly available datasets
- A description of any restrictions on data availability
- For clinical datasets or third party data, please ensure that the statement adheres to our policy

The RNA-seq data and CUT&RUN-seq data generated in this study have been deposited in the National Center for Biotechnology Information Gene Expression Omnibus (GEO) database under accession number GSE270434. Raw files for the H3Q5his mass spectrometry proteomics data in 293T cells, as well as the mass spectrometry proteomics data have been deposited to the ProteomeXchange Consortium via the PRIDE partner repository with the dataset identifiers PXD053429 and PXD053788. The atomic coordinates and structure factors have been deposited in the Protein Data Bank (PDB) under PDB ID code 8HMX. We declare that the data supporting findings for this study are available within the article and Supplementary Information. Related data are available from the corresponding author upon reasonable request. No restrictions on data availability apply.

Human research participants

Policy information about studies involving human research participants and Sex and Gender in Research.

Reporting on sex and gender	N/A
Population characteristics	N/A
Recruitment	N/A
Ethics oversight	N/A

Note that full information on the approval of the study protocol must also be provided in the manuscript.

Field-specific reporting

Please select the one below that is the best fit for your research. If you are not sure, read the appropriate sections before making your selection.

☒ Life sciences ☐ Behavioural & social sciences ☐ Ecological, evolutionary & environmental sciences

For a reference copy of the document with all sections, see nature.com/documents/nr-reporting-summary-flat.pdf

Life sciences study design

All studies must disclose on these points even when the disclosure is negative.

Sample size	Adequate sample sizes are generally determined based upon inter-sample variability. Throughout the manuscript, we determined the significance of results based upon a general confidence interval of 95%. We do not include specific justifications of sample size within the methods (e.g., power analyses), as sample sizes were based on extensive laboratory experience with these endpoints. The sample sizes chosen are consistent with those used by others in the field to achieve statistically significant results comparing stressed vs. control animals.
Data exclusions	No data exclusions.
Replication	All biological endpoints were reliably reproduced using numerous biological (>3 for all experiments in which statistics were employed) and technical replicates for each experiment. All novel reagents used in this study were extensively validated, as demonstrated in the manuscript submission.
Randomization	Where appropriate, animals were randomly assigned to groups (segregated by viral treatments, or ZT). Tissue samples were not pooled from multiple animals in these studies for western-blotting and RNA-seq experiments (i.e., each n represents a discrete data point). For CUT&RUN, each replicate per antibody was pooled from punches from 3 animals (per n) for initial processing, with 3 independent biological replicates conducted (n=3) per antibody.
Blinding	For all viral experiments (RNA-seq, western blotting and behavior), investigators were blinded to conditions such viral treatment etc. prior to analysis.

Reporting for specific materials, systems and methods

We require information from authors about some types of materials, experimental systems and methods used in many studies. Here, indicate whether each material, system or method listed is relevant to your study. If you are not sure if a list item applies to your research, read the appropriate section before selecting a response.

Materials & experimental systems

n/a	Involved in the study	
☐	☒	Antibodies
☐	☒	Eukaryotic cell lines
☒	☐	Palaeontology and archaeology
☐	☒	Animals and other organisms
☒	☐	Clinical data
☒	☐	Dual use research of concern

Methods

n/a	Involved in the study	
☐	☒	ChIP-seq
☒	☐	Flow cytometry
☒	☐	MRI-based neuroimaging

Antibodies

Antibodies used

Primary antibodies used in this study:
Rabbit Anti-TGM2 1:500 (brain) Abcam
CUB 7402
Rabbit Anti-GAPDH 1:1000 (Western) Thermo-Fisher
PA1-16777
1: 1000 (in vitro and in cellulo) CST
(3557S)
Chicken Anti-H3 1: 1000 Abcam
(ab134198)
Mouse Anti-H3 1: 1000 Abcam
(ab10799)
Mouse Anti-Actin 1: 1000 CST
(3700S)
Rabbit Anti-H3Q5ser 1: 1000 Millipore
(ABE1791)
Rabbit Anti-H3Q5dop 1: 1000 Millipore
(ABE2588)
Rabbit Anti-H3Q5his 1: 200 (brain)
1:1000 (in vitro and in cellulo) Millipore
(ABE2578)
Rabbit Anti-H3K4me3Q5ser 1:50 (CUT&RUN), 1:500 (Western) Millipore
(ABE2605)
Rabbit Anti-H3K4me3Q5his 1:50 (CUT&RUN), 1:500 (Western) Millipore
(ABE2570)
Rabbit Anti-H3K4me3 1:1000 Abcam
(ab8580)

Rabbit Anti-H3K4me3 1:50 (CUT&RUN), 1:500 (Western) Epicypher
(13-0041)
Rabbit Anti-H3K4me2 1:1000 Abcam
(ab7766)
Rabbit Anti-H3K4me2 1:50 (CUT&RUN) Active Motif
(39141)
Rabbit Anti-H3K27ac 1:1000 Active Motif
(AB_2614979)
Rabbit Anti-H3 1:50000 (brain) Abcam
(ab1791)
Rabbit Anti-WDR5 1:50 (CUT&RUN) CST
(13105)
Rabbit Anti-WDR5 1:1000 Abcam
(ab307664)
Mouse Anti-FLAG M2 1:1000 Millipore Sigma
(F1804)
Chicken Anti-6XHIS 1:1000 Invitrogen
(PA19531)
Rabbit Anti-HDC 1:1000 ARP
(03–16045)
Rabbit Anti-NeuN 1:1000 Millipore Sigma
(MAB377)

Donkey Anti-Chicken IRDye 800CW 1: 15000 (Li-Cor 926-32218)
Goat anti-Chicken IgY (H+L) Secondary Antibody, Alexa Fluor™ 488, 1:500 Invitrogen (for H3 peptide quantification) (Thermo Fisher A-11039)
Goat Anti-Mouse IRDye 680RD 1: 15000 (Li-Cor 926-68070)
Goat Anti-Mouse IRDye 800CW 1: 15000 (Li-Cor 926-32210)
Goat Anti-Rabbit IRDye 800CW 1: 15000 (Li-Cor 926-32211)
Goat Anti-Rabbit IRDye 680RD 1: 15000 (Li-Cor 926-68071)
Goat Anti-Rabbit Horseradish Peroxidase 1:10000
1:50000 (for anti-H3 antibody) (BioRad 1706515)
Sheep Anti-Mouse Horseradish Peroxidase 1:5000 (Cytiva RPN4201)
Donkey Anti-Rabbit Horseradish Peroxidase 1:5000 (Cytiva NA934)

Validation

All antibodies used in this study (all of which have been commercially validated (see manufacturers website) or described in previous publications) were validated in cells/tissues via immunoblotting, IPs or ICC/IHC/IF prior to experimentation.
Validation of all primary antibodies was additionally provided according to the manufacturer`s website/datasheet data. All information regarding validation of histone monoaminylation primary antibodies is included in the manuscript and in prior publications (cited in the manuscript).

Eukaryotic cell lines

Policy information about cell lines and Sex and Gender in Research

Cell line source(s)	HeLa (CRM-CCL-2) and HEK293T (CRL-3216) cell lines were obtained from the American Type Culture Collection (ATCC).
Authentication	Human tissue culture cell lines (HeLa, HEK293T) were imaged for appropriate morphology.
Mycoplasma contamination	We can confirm that all cell lines tested negative for mycoplasma contamination.
Commonly misidentified lines (See ICLAC register)	No commonly misidentified cell lines were used.

Animals and other research organisms

Policy information about studies involving animals; ARRIVE guidelines recommended for reporting animal research, and Sex and Gender in Research

Laboratory animals	Mice (C57BL/6J) were purchased from The Jackson Laboratory. Animals were group housed (2-5 per cage) on a 12-hour light/dark cycle (lights on from 7:00 A.M. to 7:00 P.M.) at constant temperature (23ºC) and with controlled humidity (50%) and ad libitum access to food and water. All mice used for the experiments in this manuscript were aged 8-14 weeks old. All animal protocols were approved by the IACUC at the Icahn School of Medicine at Mount Sinai (ISMMS).
Wild animals	No wild animals were used in this study.
Reporting on sex	Both male and female mice were used in this study.
Field-collected samples	No field collected samples were used in this study.
Ethics oversight	All animal protocols were approved by the IACUC at both the Icahn School of Medicine at Mount Sinai (ISMMS).

Note that full information on the approval of the study protocol must also be provided in the manuscript.

ChIP-seq

Data deposition

☒ Confirm that both raw and final processed data have been deposited in a public database such as GEO.

☒ Confirm that you have deposited or provided access to graph files (e.g. BED files) for the called peaks.

Data access links
May remain private before publication.

The CUT&RUN-seq data generated in this study have been deposited in the National Center for Biotechnology Information Gene Expression Omnibus (GEO) database under accession number GSE270434. This includes the raw fastq files for each experiment, as well as bigwigs made from merged BAM files (merging the 3 biological replicates). These bigwigs were scaled according to an E. coli spike-in DNA for each CUT&RUN reaction, with scaling happening across time-points or treatments for the same antibody. See methods for details. MACS2 peak files (.broadPeak and .narrowPeak) are available in the GEO as well for certain time points/antibodies.

Files in database submission

processed data file processed data file raw file raw file raw file raw file
0_K2.scaled.bs10.bw K4me2_ZT0_IgG.BROAD.01_peaks.broadPeak 0_K2_1_S11_L001_R1_001.fastq.gz
0_K2_1_S11_L001_R2_001.fastq.gz 0_K2_1_S11_L002_R1_001.fastq.gz 0_K2_1_S11_L002_R2_001.fastq.gz
0_K2.scaled.bs10.bw K4me2_ZT0_IgG.BROAD.01_peaks.broadPeak 0_K2_2_S12_L001_R1_001.fastq.gz
0_K2_2_S12_L001_R2_001.fastq.gz 0_K2_2_S12_L002_R1_001.fastq.gz 0_K2_2_S12_L002_R2_001.fastq.gz
0_K2.scaled.bs10.bw K4me2_ZT0_IgG.BROAD.01_peaks.broadPeak 0_K2_3_S13_L001_R1_001.fastq.gz
0_K2_3_S13_L001_R2_001.fastq.gz 0_K2_3_S13_L002_R1_001.fastq.gz 0_K2_3_S13_L002_R2_001.fastq.gz
4_K2.scaled.bs10.bw 4_K2_1_S17_L001_R1_001.fastq.gz 4_K2_1_S17_L001_R2_001.fastq.gz
4_K2_1_S17_L002_R1_001.fastq.gz 4_K2_1_S17_L002_R2_001.fastq.gz
4_K2.scaled.bs10.bw 4_K2_2_S18_L001_R1_001.fastq.gz 4_K2_2_S18_L001_R2_001.fastq.gz
4_K2_2_S18_L002_R1_001.fastq.gz 4_K2_2_S18_L002_R2_001.fastq.gz
4_K2.scaled.bs10.bw 4_K2_3_S19_L001_R1_001.fastq.gz 4_K2_3_S19_L001_R2_001.fastq.gz
4_K2_3_S19_L002_R1_001.fastq.gz 4_K2_3_S19_L002_R2_001.fastq.gz
8_K2.scaled.bs10.bw 8_K2_1_S23_L001_R1_001.fastq.gz 8_K2_1_S23_L001_R2_001.fastq.gz
8_K2_1_S23_L002_R1_001.fastq.gz 8_K2_1_S23_L002_R2_001.fastq.gz
8_K2.scaled.bs10.bw 8_K2_2_S24_L001_R1_001.fastq.gz 8_K2_2_S24_L001_R2_001.fastq.gz
8_K2_2_S24_L002_R1_001.fastq.gz 8_K2_2_S24_L002_R2_001.fastq.gz
8_K2.scaled.bs10.bw 8_K2_3_S25_L001_R1_001.fastq.gz 8_K2_3_S25_L001_R2_001.fastq.gz
8_K2_3_S25_L002_R1_001.fastq.gz 8_K2_3_S25_L002_R2_001.fastq.gz
12_K2.scaled.bs10.bw 12_K2_1_S29_L001_R1_001.fastq.gz 12_K2_1_S29_L001_R2_001.fastq.gz
12_K2_1_S29_L002_R1_001.fastq.gz 12_K2_1_S29_L002_R2_001.fastq.gz
12_K2.scaled.bs10.bw 12_K2_2_S30_L001_R1_001.fastq.gz 12_K2_2_S30_L001_R2_001.fastq.gz
12_K2_2_S30_L002_R1_001.fastq.gz 12_K2_2_S30_L002_R2_001.fastq.gz
12_K2.scaled.bs10.bw 12_K2_3_S31_L001_R1_001.fastq.gz 12_K2_3_S31_L001_R2_001.fastq.gz
12_K2_3_S31_L002_R1_001.fastq.gz 12_K2_3_S31_L002_R2_001.fastq.gz
16_K2.scaled.bs10.bw 16_K2_1_S35_L001_R1_001.fastq.gz 16_K2_1_S35_L001_R2_001.fastq.gz
16_K2_1_S35_L002_R1_001.fastq.gz 16_K2_1_S35_L002_R2_001.fastq.gz
16_K2.scaled.bs10.bw 16_K2_2_S36_L001_R1_001.fastq.gz 16_K2_2_S36_L001_R2_001.fastq.gz
16_K2_2_S36_L002_R1_001.fastq.gz 16_K2_2_S36_L002_R2_001.fastq.gz
16_K2.scaled.bs10.bw 16_K2_3_S37_L001_R1_001.fastq.gz 16_K2_3_S37_L001_R2_001.fastq.gz
16_K2_3_S37_L002_R1_001.fastq.gz 16_K2_3_S37_L002_R2_001.fastq.gz
20_K2.scaled.bs10.bw 20_K2_1_S41_L001_R1_001.fastq.gz 20_K2_1_S41_L001_R2_001.fastq.gz
20_K2_1_S41_L002_R1_001.fastq.gz 20_K2_1_S41_L002_R2_001.fastq.gz
20_K2.scaled.bs10.bw 20_K2_2_S42_L001_R1_001.fastq.gz 20_K2_2_S42_L001_R2_001.fastq.gz
20_K2_2_S42_L002_R1_001.fastq.gz 20_K2_2_S42_L002_R2_001.fastq.gz
20_K2.scaled.bs10.bw 20_K2_3_S43_L001_R1_001.fastq.gz 20_K2_3_S43_L001_R2_001.fastq.gz
20_K2_3_S43_L002_R1_001.fastq.gz 20_K2_3_S43_L002_R2_001.fastq.gz
0_K3.scaled.bs10.bw K4me3_ZT0_IgG.BROAD.05_peaks.broadPeak 0_K3_1_S14_L001_R1_001.fastq.gz
0_K3_1_S14_L001_R2_001.fastq.gz 0_K3_1_S14_L002_R1_001.fastq.gz 0_K3_1_S14_L002_R2_001.fastq.gz
0_K3.scaled.bs10.bw K4me3_ZT0_IgG.BROAD.05_peaks.broadPeak 0_K3_2_S15_L001_R1_001.fastq.gz
0_K3_2_S15_L001_R2_001.fastq.gz 0_K3_2_S15_L002_R1_001.fastq.gz 0_K3_2_S15_L002_R2_001.fastq.gz
0_K3.scaled.bs10.bw K4me3_ZT0_IgG.BROAD.05_peaks.broadPeak 0_K3_3_S16_L001_R1_001.fastq.gz
0_K3_3_S16_L001_R2_001.fastq.gz 0_K3_3_S16_L002_R1_001.fastq.gz 0_K3_3_S16_L002_R2_001.fastq.gz
4_K3.scaled.bs10.bw 4_K3_1_S20_L001_R1_001.fastq.gz 4_K3_1_S20_L001_R2_001.fastq.gz
4_K3_1_S20_L002_R1_001.fastq.gz 4_K3_1_S20_L002_R2_001.fastq.gz
4_K3.scaled.bs10.bw 4_K3_2_S21_L001_R1_001.fastq.gz 4_K3_2_S21_L001_R2_001.fastq.gz
4_K3_2_S21_L002_R1_001.fastq.gz 4_K3_2_S21_L002_R2_001.fastq.gz
4_K3.scaled.bs10.bw 4_K3_3_S22_L001_R1_001.fastq.gz 4_K3_3_S22_L001_R2_001.fastq.gz
4_K3_3_S22_L002_R1_001.fastq.gz 4_K3_3_S22_L002_R2_001.fastq.gz
8_K3.scaled.bs10.bw 8_K3_1_S26_L001_R1_001.fastq.gz 8_K3_1_S26_L001_R2_001.fastq.gz
8_K3_1_S26_L002_R1_001.fastq.gz 8_K3_1_S26_L002_R2_001.fastq.gz
8_K3.scaled.bs10.bw 8_K3_2_S27_L001_R1_001.fastq.gz 8_K3_2_S27_L001_R2_001.fastq.gz
8_K3_2_S27_L002_R1_001.fastq.gz 8_K3_2_S27_L002_R2_001.fastq.gz
8_K3.scaled.bs10.bw 8_K3_3_S28_L001_R1_001.fastq.gz 8_K3_3_S28_L001_R2_001.fastq.gz
8_K3_3_S28_L002_R1_001.fastq.gz 8_K3_3_S28_L002_R2_001.fastq.gz
12_K3.scaled.bs10.bw 12_K3_1_S32_L001_R1_001.fastq.gz 12_K3_1_S32_L001_R2_001.fastq.gz
12_K3_1_S32_L002_R1_001.fastq.gz 12_K3_1_S32_L002_R2_001.fastq.gz
12_K3.scaled.bs10.bw 12_K3_2_S33_L001_R1_001.fastq.gz 12_K3_2_S33_L001_R2_001.fastq.gz
12_K3_2_S33_L002_R1_001.fastq.gz 12_K3_2_S33_L002_R2_001.fastq.gz

12_K3.scaled.bs10.bw 12_K3_3_S34_L001_R1_001.fastq.gz 12_K3_3_S34_L001_R2_001.fastq.gz
12_K3_3_S34_L002_R1_001.fastq.gz 12_K3_3_S34_L002_R2_001.fastq.gz
16_K3.scaled.bs10.bw 16_K3_1_S38_L001_R1_001.fastq.gz 16_K3_1_S38_L001_R2_001.fastq.gz
16_K3_1_S38_L002_R1_001.fastq.gz 16_K3_1_S38_L002_R2_001.fastq.gz
16_K3.scaled.bs10.bw 16_K3_2_S39_L001_R1_001.fastq.gz 16_K3_2_S39_L001_R2_001.fastq.gz
16_K3_2_S39_L002_R1_001.fastq.gz 16_K3_2_S39_L002_R2_001.fastq.gz
16_K3.scaled.bs10.bw 16_K3_3_S40_L001_R1_001.fastq.gz 16_K3_3_S40_L001_R2_001.fastq.gz
16_K3_3_S40_L002_R1_001.fastq.gz 16_K3_3_S40_L002_R2_001.fastq.gz
20_K3.scaled.bs10.bw 20_K3_1_S44_L001_R1_001.fastq.gz 20_K3_1_S44_L001_R2_001.fastq.gz
20_K3_1_S44_L002_R1_001.fastq.gz 20_K3_1_S44_L002_R2_001.fastq.gz
20_K3.scaled.bs10.bw 20_K3_2_S45_L001_R1_001.fastq.gz 20_K3_2_S45_L001_R2_001.fastq.gz
20_K3_2_S45_L002_R1_001.fastq.gz 20_K3_2_S45_L002_R2_001.fastq.gz
20_K3.scaled.bs10.bw 20_K3_3_S46_L001_R1_001.fastq.gz 20_K3_3_S46_L001_R2_001.fastq.gz
20_K3_3_S46_L002_R1_001.fastq.gz 20_K3_3_S46_L002_R2_001.fastq.gz
0_DH_merged.scaled.bs10.bw 0_DH_1_S29_L001_R1_001.fastq.gz 0_DH_1_S29_L001_R2_001.fastq.gz
0_DH_1_S29_L002_R1_001.fastq.gz 0_DH_1_S29_L002_R2_001.fastq.gz
0_DH_merged.scaled.bs10.bw 0_DH_2_S30_L001_R1_001.fastq.gz 0_DH_2_S30_L001_R2_001.fastq.gz
0_DH_2_S30_L002_R1_001.fastq.gz 0_DH_2_S30_L002_R2_001.fastq.gz
0_DH_merged.scaled.bs10.bw 0_DH_3_S31_L001_R1_001.fastq.gz 0_DH_3_S31_L001_R2_001.fastq.gz
0_DH_3_S31_L002_R1_001.fastq.gz 0_DH_3_S31_L002_R2_001.fastq.gz
4_DH.scaled.bs10.bw 4_DH_1_S1_L001_R1_001.fastq.gz 4_DH_1_S1_L001_R2_001.fastq.gz
4_DH_1_S1_L002_R1_001.fastq.gz 4_DH_1_S1_L002_R2_001.fastq.gz
4_DH.scaled.bs10.bw 4_DH_2_S2_L001_R1_001.fastq.gz 4_DH_2_S2_L001_R2_001.fastq.gz
4_DH_2_S2_L002_R1_001.fastq.gz 4_DH_2_S2_L002_R2_001.fastq.gz
4_DH.scaled.bs10.bw 4_DH_3_S3_L001_R1_001.fastq.gz 4_DH_3_S3_L001_R2_001.fastq.gz
4_DH_3_S3_L002_R1_001.fastq.gz 4_DH_3_S3_L002_R2_001.fastq.gz
8_DH_merged.scaled.bs10.bw 8_DH_1_S49_L001_R1_001.fastq.gz 8_DH_1_S49_L001_R2_001.fastq.gz
8_DH_1_S49_L002_R1_001.fastq.gz 8_DH_1_S49_L002_R2_001.fastq.gz
8_DH_merged.scaled.bs10.bw 8_DH_2_S50_L001_R1_001.fastq.gz 8_DH_2_S50_L001_R2_001.fastq.gz
8_DH_2_S50_L002_R1_001.fastq.gz 8_DH_2_S50_L002_R2_001.fastq.gz
8_DH_merged.scaled.bs10.bw 8_DH_3_S51_L001_R1_001.fastq.gz 8_DH_3_S51_L001_R2_001.fastq.gz
8_DH_3_S51_L002_R1_001.fastq.gz 8_DH_3_S51_L002_R2_001.fastq.gz
12_DH_merged.scaled.bs10.bw 12_DH_1_S59_L001_R1_001.fastq.gz 12_DH_1_S59_L001_R2_001.fastq.gz
12_DH_1_S59_L002_R1_001.fastq.gz 12_DH_1_S59_L002_R2_001.fastq.gz
12_DH_merged.scaled.bs10.bw 12_DH_2_S60_L001_R1_001.fastq.gz 12_DH_2_S60_L001_R2_001.fastq.gz
12_DH_2_S60_L002_R1_001.fastq.gz 12_DH_2_S60_L002_R2_001.fastq.gz
12_DH_merged.scaled.bs10.bw 12_DH_3_S61_L001_R1_001.fastq.gz 12_DH_3_S61_L001_R2_001.fastq.gz
12_DH_3_S61_L002_R1_001.fastq.gz 12_DH_3_S61_L002_R2_001.fastq.gz
16_DH_merged.scaled.bs10.bw DualHis_ZT16_IgG.BROAD.05_peaks.broadPeak 16_DH_1_S69_L001_R1_001.fastq.gz
16_DH_1_S69_L001_R2_001.fastq.gz 16_DH_1_S69_L002_R1_001.fastq.gz 16_DH_1_S69_L002_R2_001.fastq.gz
16_DH_merged.scaled.bs10.bw DualHis_ZT16_IgG.BROAD.05_peaks.broadPeak 16_DH_2_S70_L001_R1_001.fastq.gz
16_DH_2_S70_L001_R2_001.fastq.gz 16_DH_2_S70_L002_R1_001.fastq.gz 16_DH_2_S70_L002_R2_001.fastq.gz
16_DH_merged.scaled.bs10.bw DualHis_ZT16_IgG.BROAD.05_peaks.broadPeak 16_DH_3_S71_L001_R1_001.fastq.gz
16_DH_3_S71_L001_R2_001.fastq.gz 16_DH_3_S71_L002_R1_001.fastq.gz 16_DH_3_S71_L002_R2_001.fastq.gz
20_DH_merged.scaled.bs10.bw 20_DH_1_S79_L001_R1_001.fastq.gz 20_DH_1_S79_L001_R2_001.fastq.gz
20_DH_1_S79_L002_R1_001.fastq.gz 20_DH_1_S79_L002_R2_001.fastq.gz
20_DH_merged.scaled.bs10.bw 20_DH_2_S80_L001_R1_001.fastq.gz 20_DH_2_S80_L001_R2_001.fastq.gz
20_DH_2_S80_L002_R1_001.fastq.gz 20_DH_2_S80_L002_R2_001.fastq.gz
20_DH_merged.scaled.bs10.bw 20_DH_3_S81_L001_R1_001.fastq.gz 20_DH_3_S81_L001_R2_001.fastq.gz
20_DH_3_S81_L002_R1_001.fastq.gz 20_DH_3_S81_L002_R2_001.fastq.gz
0_DS_merged.scaled.bs10.bw 0_DS_1_S32_L001_R1_001.fastq.gz 0_DS_1_S32_L001_R2_001.fastq.gz
0_DS_1_S32_L002_R1_001.fastq.gz 0_DS_1_S32_L002_R2_001.fastq.gz
0_DS_merged.scaled.bs10.bw 0_DS_2_S33_L001_R1_001.fastq.gz 0_DS_2_S33_L001_R2_001.fastq.gz
0_DS_2_S33_L002_R1_001.fastq.gz 0_DS_2_S33_L002_R2_001.fastq.gz
0_DS_merged.scaled.bs10.bw 0_DS_3_S34_L001_R1_001.fastq.gz 0_DS_3_S34_L001_R2_001.fastq.gz
0_DS_3_S34_L002_R1_001.fastq.gz 0_DS_3_S34_L002_R2_001.fastq.gz
4_DS.scaled.bs10.bw 4_DS_1_S4_L001_R1_001.fastq.gz 4_DS_1_S4_L001_R2_001.fastq.gz
4_DS_1_S4_L002_R1_001.fastq.gz 4_DS_1_S4_L002_R2_001.fastq.gz
4_DS.scaled.bs10.bw 4_DS_2_S5_L001_R1_001.fastq.gz 4_DS_2_S5_L001_R2_001.fastq.gz
4_DS_2_S5_L002_R1_001.fastq.gz 4_DS_2_S5_L002_R2_001.fastq.gz
4_DS.scaled.bs10.bw 4_DS_3_S6_L001_R1_001.fastq.gz 4_DS_3_S6_L001_R2_001.fastq.gz
4_DS_3_S6_L002_R1_001.fastq.gz 4_DS_3_S6_L002_R2_001.fastq.gz
8_DS_merged.scaled.bs10.bw 8_DS_1_S52_L001_R1_001.fastq.gz 8_DS_1_S52_L001_R2_001.fastq.gz
8_DS_1_S52_L002_R1_001.fastq.gz 8_DS_1_S52_L002_R2_001.fastq.gz
8_DS_merged.scaled.bs10.bw 8_DS_2_S53_L001_R1_001.fastq.gz 8_DS_2_S53_L001_R2_001.fastq.gz
8_DS_2_S53_L002_R1_001.fastq.gz 8_DS_2_S53_L002_R2_001.fastq.gz
8_DS_merged.scaled.bs10.bw 8_DS_3_S54_L001_R1_001.fastq.gz 8_DS_3_S54_L001_R2_001.fastq.gz
8_DS_3_S54_L002_R1_001.fastq.gz 8_DS_3_S54_L002_R2_001.fastq.gz
12_DS_merged.scaled.bs10.bw 12_DS_1_S62_L001_R1_001.fastq.gz 12_DS_1_S62_L001_R2_001.fastq.gz
12_DS_1_S62_L002_R1_001.fastq.gz 12_DS_1_S62_L002_R2_001.fastq.gz
12_DS_merged.scaled.bs10.bw 12_DS_2_S63_L001_R1_001.fastq.gz 12_DS_2_S63_L001_R2_001.fastq.gz
12_DS_2_S63_L002_R1_001.fastq.gz 12_DS_2_S63_L002_R2_001.fastq.gz
12_DS_merged.scaled.bs10.bw 12_DS_3_S64_L001_R1_001.fastq.gz 12_DS_3_S64_L001_R2_001.fastq.gz
12_DS_3_S64_L002_R1_001.fastq.gz 12_DS_3_S64_L002_R2_001.fastq.gz
16_DS_merged.scaled.bs10.bw 16_DS_1_S72_L001_R1_001.fastq.gz 16_DS_1_S72_L001_R2_001.fastq.gz
16_DS_1_S72_L002_R1_001.fastq.gz 16_DS_1_S72_L002_R2_001.fastq.gz

16_DS_merged.scaled.bs10.bw 16_DS_2_S73_L001_R1_001.fastq.gz 16_DS_2_S73_L001_R2_001.fastq.gz
16_DS_2_S73_L002_R1_001.fastq.gz 16_DS_2_S73_L002_R2_001.fastq.gz
16_DS_merged.scaled.bs10.bw 16_DS_3_S74_L001_R1_001.fastq.gz 16_DS_3_S74_L001_R2_001.fastq.gz
16_DS_3_S74_L002_R1_001.fastq.gz 16_DS_3_S74_L002_R2_001.fastq.gz
20_DS_merged.scaled.bs10.bw 20_DS_1_S82_L001_R1_001.fastq.gz 20_DS_1_S82_L001_R2_001.fastq.gz
20_DS_1_S82_L002_R1_001.fastq.gz 20_DS_1_S82_L002_R2_001.fastq.gz
20_DS_merged.scaled.bs10.bw 20_DS_2_S83_L001_R1_001.fastq.gz 20_DS_2_S83_L001_R2_001.fastq.gz
20_DS_2_S83_L002_R1_001.fastq.gz 20_DS_2_S83_L002_R2_001.fastq.gz
20_DS_merged.scaled.bs10.bw 20_DS_3_S84_L001_R1_001.fastq.gz 20_DS_3_S84_L001_R2_001.fastq.gz
20_DS_3_S84_L002_R1_001.fastq.gz 20_DS_3_S84_L002_R2_001.fastq.gz
0_WD_merged.scaled.bs10.bw 0_WD_1_S42_L001_R1_001.fastq.gz 0_WD_1_S42_L001_R2_001.fastq.gz
0_WD_1_S42_L002_R1_001.fastq.gz 0_WD_1_S42_L002_R2_001.fastq.gz
0_WD_merged.scaled.bs10.bw 0_WD_2_S43_L001_R1_001.fastq.gz 0_WD_2_S43_L001_R2_001.fastq.gz
0_WD_2_S43_L002_R1_001.fastq.gz 0_WD_2_S43_L002_R2_001.fastq.gz
0_WD_merged.scaled.bs10.bw 0_WD_3_S37_L001_R1_001.fastq.gz 0_WD_3_S37_L001_R2_001.fastq.gz
0_WD_3_S37_L002_R1_001.fastq.gz 0_WD_3_S37_L002_R2_001.fastq.gz
4_WD.scaled.bs10.bw 4_WD_1_S7_L001_R1_001.fastq.gz 4_WD_1_S7_L001_R2_001.fastq.gz
4_WD_1_S7_L002_R1_001.fastq.gz 4_WD_1_S7_L002_R2_001.fastq.gz
4_WD.scaled.bs10.bw 4_WD_2_S8_L001_R1_001.fastq.gz 4_WD_2_S8_L001_R2_001.fastq.gz
4_WD_2_S8_L002_R1_001.fastq.gz 4_WD_2_S8_L002_R2_001.fastq.gz
4_WD.scaled.bs10.bw 4_WD_3_S9_L001_R1_001.fastq.gz 4_WD_3_S9_L001_R2_001.fastq.gz
4_WD_3_S9_L002_R1_001.fastq.gz 4_WD_3_S9_L002_R2_001.fastq.gz
8_WD_merged.scaled.bs10.bw 8_WD_1_S55_L001_R1_001.fastq.gz 8_WD_1_S55_L001_R2_001.fastq.gz
8_WD_1_S55_L002_R1_001.fastq.gz 8_WD_1_S55_L002_R2_001.fastq.gz
8_WD_merged.scaled.bs10.bw 8_WD_2_S56_L001_R1_001.fastq.gz 8_WD_2_S56_L001_R2_001.fastq.gz
8_WD_2_S56_L002_R1_001.fastq.gz 8_WD_2_S56_L002_R2_001.fastq.gz
8_WD_merged.scaled.bs10.bw 8_WD_3_S57_L001_R1_001.fastq.gz 8_WD_3_S57_L001_R2_001.fastq.gz
8_WD_3_S57_L002_R1_001.fastq.gz 8_WD_3_S57_L002_R2_001.fastq.gz
12_WD_merged.scaled.bs10.bw 12_WD_1_S65_L001_R1_001.fastq.gz 12_WD_1_S65_L001_R2_001.fastq.gz
12_WD_1_S65_L002_R1_001.fastq.gz 12_WD_1_S65_L002_R2_001.fastq.gz
12_WD_merged.scaled.bs10.bw 12_WD_2_S66_L001_R1_001.fastq.gz 12_WD_2_S66_L001_R2_001.fastq.gz
12_WD_2_S66_L002_R1_001.fastq.gz 12_WD_2_S66_L002_R2_001.fastq.gz
12_WD_merged.scaled.bs10.bw 12_WD_3_S67_L001_R1_001.fastq.gz 12_WD_3_S67_L001_R2_001.fastq.gz
12_WD_3_S67_L002_R1_001.fastq.gz 12_WD_3_S67_L002_R2_001.fastq.gz
16_WD_merged.scaled.bs10.bw WDR5_16_IgG.05_peaks.narrowPeak 16_WD_1_S75_L001_R1_001.fastq.gz
16_WD_1_S75_L001_R2_001.fastq.gz 16_WD_1_S75_L002_R1_001.fastq.gz 16_WD_1_S75_L002_R2_001.fastq.gz
16_WD_merged.scaled.bs10.bw WDR5_16_IgG.05_peaks.narrowPeak 16_WD_2_S76_L001_R1_001.fastq.gz
16_WD_2_S76_L001_R2_001.fastq.gz 16_WD_2_S76_L002_R1_001.fastq.gz 16_WD_2_S76_L002_R2_001.fastq.gz
16_WD_merged.scaled.bs10.bw WDR5_16_IgG.05_peaks.narrowPeak 16_WD_3_S77_L001_R1_001.fastq.gz
16_WD_3_S77_L001_R2_001.fastq.gz 16_WD_3_S77_L002_R1_001.fastq.gz 16_WD_3_S77_L002_R2_001.fastq.gz
20_WD_merged.scaled.bs10.bw 20_WD_1_S85_L001_R1_001.fastq.gz 20_WD_1_S85_L001_R2_001.fastq.gz
20_WD_1_S85_L002_R1_001.fastq.gz 20_WD_1_S85_L002_R2_001.fastq.gz
20_WD_merged.scaled.bs10.bw 20_WD_2_S86_L001_R1_001.fastq.gz 20_WD_2_S86_L001_R2_001.fastq.gz
20_WD_2_S86_L002_R1_001.fastq.gz 20_WD_2_S86_L002_R2_001.fastq.gz
20_WD_merged.scaled.bs10.bw 20_WD_3_S87_L001_R1_001.fastq.gz 20_WD_3_S87_L001_R2_001.fastq.gz
20_WD_3_S87_L002_R1_001.fastq.gz 20_WD_3_S87_L002_R2_001.fastq.gz
0_IgG_.scaled.bs10.bw 0_IgG_S38_L001_R1_001.fastq.gz 0_IgG_S38_L001_R2_001.fastq.gz
0_IgG_S38_L002_R1_001.fastq.gz 0_IgG_S38_L002_R2_001.fastq.gz
4_IgG.scaled.bs10.bw 4_IgG_S10_L001_R1_001.fastq.gz 4_IgG_S10_L001_R2_001.fastq.gz
4_IgG_S10_L002_R1_001.fastq.gz 4_IgG_S10_L002_R2_001.fastq.gz
8_IgG_.scaled.bs10.bw 8_IgG_S58_L001_R1_001.fastq.gz 8_IgG_S58_L001_R2_001.fastq.gz
8_IgG_S58_L002_R1_001.fastq.gz 8_IgG_S58_L002_R2_001.fastq.gz
12_IgG_.scaled.bs10.bw 12_IgG_S68_L001_R1_001.fastq.gz 12_IgG_S68_L001_R2_001.fastq.gz
12_IgG_S68_L002_R1_001.fastq.gz 12_IgG_S68_L002_R2_001.fastq.gz
16_IgG_.scaled.bs10.bw 16_IgG_S78_L001_R1_001.fastq.gz 16_IgG_S78_L001_R2_001.fastq.gz
16_IgG_S78_L002_R1_001.fastq.gz 16_IgG_S78_L002_R2_001.fastq.gz
20_IgG_.scaled.bs10.bw 20_IgG_S88_L001_R1_001.fastq.gz 20_IgG_S88_L001_R2_001.fastq.gz
20_IgG_S88_L002_R1_001.fastq.gz 20_IgG_S88_L002_R2_001.fastq.gz
V_WD.scaled.bs10.bw V_WD_1_S53_L001_R1_001.fastq.gz V_WD_1_S53_L001_R2_001.fastq.gz
V_WD_1_S53_L002_R1_001.fastq.gz V_WD_1_S53_L002_R2_001.fastq.gz
V_WD.scaled.bs10.bw V_WD_2_S54_L001_R1_001.fastq.gz V_WD_2_S54_L001_R2_001.fastq.gz
V_WD_2_S54_L002_R1_001.fastq.gz V_WD_2_S54_L002_R2_001.fastq.gz
V_WD.scaled.bs10.bw V_WD_3_S55_L001_R1_001.fastq.gz V_WD_3_S55_L001_R2_001.fastq.gz
V_WD_3_S55_L002_R1_001.fastq.gz V_WD_3_S55_L002_R2_001.fastq.gz
Z_WD.scaled.bs10.bw Z_WD_1_S63_L001_R1_001.fastq.gz Z_WD_1_S63_L001_R2_001.fastq.gz
Z_WD_1_S63_L002_R1_001.fastq.gz Z_WD_1_S63_L002_R2_001.fastq.gz
Z_WD.scaled.bs10.bw Z_WD_2_S64_L001_R1_001.fastq.gz Z_WD_2_S64_L001_R2_001.fastq.gz
Z_WD_2_S64_L002_R1_001.fastq.gz Z_WD_2_S64_L002_R2_001.fastq.gz
Z_WD.scaled.bs10.bw Z_WD_3_S65_L001_R1_001.fastq.gz Z_WD_3_S65_L001_R2_001.fastq.gz
Z_WD_3_S65_L002_R1_001.fastq.gz Z_WD_3_S65_L002_R2_001.fastq.gz
V_DH.scaled.bs10.bw V_DH_1_S47_L001_R1_001.fastq.gz V_DH_1_S47_L001_R2_001.fastq.gz
V_DH_1_S47_L002_R1_001.fastq.gz V_DH_1_S47_L002_R2_001.fastq.gz
V_DH.scaled.bs10.bw V_DH_2_S48_L001_R1_001.fastq.gz V_DH_2_S48_L001_R2_001.fastq.gz
V_DH_2_S48_L002_R1_001.fastq.gz V_DH_2_S48_L002_R2_001.fastq.gz
V_DH.scaled.bs10.bw V_DH_3_S49_L001_R1_001.fastq.gz V_DH_3_S49_L001_R2_001.fastq.gz
V_DH_3_S49_L002_R1_001.fastq.gz V_DH_3_S49_L002_R2_001.fastq.gz

Z_DH.scaled.bs10.bw Z_DH_1_S57_L001_R1_001.fastq.gz Z_DH_1_S57_L001_R2_001.fastq.gz
Z_DH_1_S57_L002_R1_001.fastq.gz Z_DH_1_S57_L002_R2_001.fastq.gz
Z_DH.scaled.bs10.bw Z_DH_2_S58_L001_R1_001.fastq.gz Z_DH_2_S58_L001_R2_001.fastq.gz
Z_DH_2_S58_L002_R1_001.fastq.gz Z_DH_2_S58_L002_R2_001.fastq.gz
Z_DH.scaled.bs10.bw Z_DH_3_S59_L001_R1_001.fastq.gz Z_DH_3_S59_L001_R2_001.fastq.gz
Z_DH_3_S59_L002_R1_001.fastq.gz Z_DH_3_S59_L002_R2_001.fastq.gz
V_DS.scaled.bs10.bw V_DS_1_S50_L001_R1_001.fastq.gz V_DS_1_S50_L001_R2_001.fastq.gz
V_DS_1_S50_L002_R1_001.fastq.gz V_DS_1_S50_L002_R2_001.fastq.gz
V_DS.scaled.bs10.bw V_DS_2_S51_L001_R1_001.fastq.gz V_DS_2_S51_L001_R2_001.fastq.gz
V_DS_2_S51_L002_R1_001.fastq.gz V_DS_2_S51_L002_R2_001.fastq.gz
V_DS.scaled.bs10.bw V_DS_3_S52_L001_R1_001.fastq.gz V_DS_3_S52_L001_R2_001.fastq.gz
V_DS_3_S52_L002_R1_001.fastq.gz V_DS_3_S52_L002_R2_001.fastq.gz
Z_DS.scaled.bs10.bw Z_DS_1_S60_L001_R1_001.fastq.gz Z_DS_1_S60_L001_R2_001.fastq.gz
Z_DS_1_S60_L002_R1_001.fastq.gz Z_DS_1_S60_L002_R2_001.fastq.gz
Z_DS.scaled.bs10.bw Z_DS_2_S61_L001_R1_001.fastq.gz Z_DS_2_S61_L001_R2_001.fastq.gz
Z_DS_2_S61_L002_R1_001.fastq.gz Z_DS_2_S61_L002_R2_001.fastq.gz
Z_DS.scaled.bs10.bw Z_DS_3_S62_L001_R1_001.fastq.gz Z_DS_3_S62_L001_R2_001.fastq.gz
Z_DS_3_S62_L002_R1_001.fastq.gz Z_DS_3_S62_L002_R2_001.fastq.gz
V_IgG.scaled.bs10.bw V_IgG_S56_L001_R1_001.fastq.gz V_IgG_S56_L001_R2_001.fastq.gz
V_IgG_S56_L002_R1_001.fastq.gz V_IgG_S56_L002_R2_001.fastq.gz
Z_IgG.scaled.bs10.bw Z_IgG_S66_L001_R1_001.fastq.gz Z_IgG_S66_L001_R2_001.fastq.gz
Z_IgG_S66_L002_R1_001.fastq.gz Z_IgG_S66_L002_R2_001.fastq.gz
HELA_DH_merged.bs10.bw DualHisHela_.BROAD.05_peaks.broadPeak H_DH_1_S1_L001_R1_001.fastq.gz
H_DH_1_S1_L001_R2_001.fastq.gz H_DH_1_S1_L002_R1_001.fastq.gz H_DH_1_S1_L002_R2_001.fastq.gz
HELA_DH_merged.bs10.bw DualHisHela_.BROAD.05_peaks.broadPeak H_DH_2_S2_L001_R1_001.fastq.gz
H_DH_2_S2_L001_R2_001.fastq.gz H_DH_2_S2_L002_R1_001.fastq.gz H_DH_2_S2_L002_R2_001.fastq.gz
HELA_DH_merged.bs10.bw DualHisHela_.BROAD.05_peaks.broadPeak H_DH_3_S3_L001_R1_001.fastq.gz
H_DH_3_S3_L001_R2_001.fastq.gz H_DH_3_S3_L002_R1_001.fastq.gz H_DH_3_S3_L002_R2_001.fastq.gz
HELA_Q5_merged.bs10.bw singleHisHela_.BROAD.05_peaks.broadPeak H_Q5_1_S7_L001_R1_001.fastq.gz
H_Q5_1_S7_L001_R2_001.fastq.gz H_Q5_1_S7_L002_R1_001.fastq.gz H_Q5_1_S7_L002_R2_001.fastq.gz
HELA_Q5_merged.bs10.bw singleHisHela_.BROAD.05_peaks.broadPeak H_Q5_2_S8_L001_R1_001.fastq.gz
H_Q5_2_S8_L001_R2_001.fastq.gz H_Q5_2_S8_L002_R1_001.fastq.gz H_Q5_2_S8_L002_R2_001.fastq.gz
HELA_Q5_merged.bs10.bw singleHisHela_.BROAD.05_peaks.broadPeak H_Q5_3_S9_L001_R1_001.fastq.gz
H_Q5_3_S9_L001_R2_001.fastq.gz H_Q5_3_S9_L002_R1_001.fastq.gz H_Q5_3_S9_L002_R2_001.fastq.gz
H_IgG1_merged.sorted.RPKM.bs10.sl30.bw H_IgG1_S16_L001_R1_001.fastq.gz H_IgG1_S16_L001_R2_001.fastq.gz
H_IgG1_S16_L002_R1_001.fastq.gz H_IgG1_S16_L002_R2_001.fastq.gz

Genome browser session
(e.g. UCSC)

Hela: https://ramaka02.dmz.hpc.mssm.edu/UCSC-TrackHub-Ben-Weekly-hg19/
TMN: https://ramaka02.dmz.hpc.mssm.edu/UCSC-TrackHub-Ben-Weekly-mm10/

Methodology

Replicates

There were 3 independent biological replicates for each antibody and timepoint/treatment, each coming from a biologically independent pool of TMN or plate of HeLa cells. These biological replicates were assessed for similarity visually in IGV and by comparing alignment rate, peak calling (between biological replicates), and heatmaps (using MACS2/Deeptools). All replicates had high concordance.

Sequencing depth

CUT&RUN Sample Total Reads Mapped Reads Uniquely Mapped Reads
0_DH_1_S29 27,310,000.00 19,138,070.00 15,231,930.00
0_DH_2_S30 33,857,086.00 22,542,838.00 17,311,850.00
0_DH_3_S31 37,720,716.00 24,222,108.00 19,511,182.00
0_DS_1_S32 36,834,540.00 21,906,184.00 19,646,366.00
0_DS_2_S33 24,263,036.00 18,676,828.00 17,707,738.00
0_DS_3_S34 27,866,782.00 19,837,988.00 17,410,712.00
0_IgG_S38 24,838,152.00 12,933,064.00 9,756,070.00
0_WD_1_S42 145,472,050.00 83,380,212.00 60,985,598.00
0_WD_2_S36 170,384,052.00 98,318,428.00 71,122,742.00
0_WD_3_S37 35,270,882.00 19,165,556.00 13,923,846.00
8_DH_1_S49 52,269,610.00 29,710,832.00 24,397,130.00
8_DH_2_S50 36,998,278.00 30,050,604.00 24,494,390.00
8_DH_3_S51 40,419,368.00 27,696,056.00 22,443,284.00
8_DS_1_S52 50,859,580.00 29,984,662.00 28,075,652.00
8_DS_2_S53 38,031,308.00 29,442,818.00 27,775,760.00
8_DS_3_S54 35,845,198.00 21,444,614.00 20,094,144.00
8_IgG_S58 43,005,152.00 1,299,376.00 895,190.00
8_WD_1_S55 41,689,350.00 3,633,800.00 2,762,854.00
8_WD_2_S56 38,382,544.00 2,285,056.00 1,725,408.00
8_WD_3_S57 50,556,268.00 4,423,702.00 3,387,026.00
12_DH_1_S59 42,259,492.00 28,133,104.00 21,629,474.00
12_DH_2_S60 30,616,660.00 15,163,518.00 12,479,764.00
12_DH_3_S61 40,945,708.00 23,859,002.00 18,039,254.00
12_DS_1_S62 52,338,258.00 33,210,612.00 26,393,732.00
12_DS_2_S63 34,494,070.00 24,143,684.00 22,433,166.00
12_DS_3_S64 38,957,388.00 24,213,422.00 22,296,134.00
12_IgG_S68 40,479,908.00 22,168,700.00 16,662,982.00

12_WD_1_S65	39,362,640.00	14,189,274.00	10,815,136.00
12_WD_2_S66	43,578,482.00	7,697,774.00	5,919,686.00
12_WD_3_S67	27,574,516.00	4,293,534.00	3,495,976.00
16_DH_1_S69	33,757,586.00	29,361,130.00	23,752,622.00
16_DH_2_S70	33,344,202.00	29,017,722.00	23,642,692.00
16_DH_3_S71	30,410,508.00	26,450,556.00	21,805,204.00
16_DS_1_S72	36,357,196.00	30,456,338.00	28,877,942.00
16_DS_2_S73	29,347,110.00	26,841,350.00	25,421,054.00
16_DS_3_S74	30,114,608.00	23,679,988.00	22,187,514.00
16_IgG_S78	25,302,004.00	5,882,086.00	4,731,038.00
16_WD_1_S75	31,995,666.00	13,152,232.00	11,088,520.00
16_WD_2_S76	30,265,902.00	11,832,936.00	10,303,224.00
16_WD_3_S77	28,633,122.00	7,582,756.00	6,467,248.00
20_DH_1_S79	21,578,164.00	18,400,694.00	14,638,490.00
20_DH_2_S80	34,264,656.00	28,928,486.00	24,103,634.00
20_DH_3_S81	29,568,680.00	27,069,202.00	22,049,074.00
20_DS_1_S82	37,368,162.00	33,076,776.00	31,023,900.00
20_DS_2_S83	26,879,130.00	23,250,150.00	21,905,280.00
20_DS_3_S84	28,888,342.00	23,754,990.00	22,413,478.00
20_IgG_S88	34,850,010.00	5,608,916.00	4,374,330.00
20_WD_1_S85	22,031,330.00	4,625,340.00	3,953,072.00
20_WD_2_S86	33,663,820.00	3,749,416.00	3,002,986.00
20_WD_3_S87	28,524,766.00	6,099,688.00	4,759,672.00
0_K2_1_S11	38,592,784.00	30,304,078.00	28,372,604.00
0_K2_2_S12	50,108,656.00	37,949,908.00	35,239,812.00
0_K2_3_S13	60,436,514.00	42,626,340.00	39,164,730.00
0_K3_1_S14	46,896,318.00	21,782,728.00	17,700,696.00
0_K3_2_S15	46,494,206.00	22,304,170.00	18,191,010.00
0_K3_3_S16	44,252,650.00	20,464,314.00	16,619,598.00
4_DH_1_S1	49,322,612.00	5,530,438.00	4,598,906.00
4_DH_2_S2	51,945,794.00	7,297,390.00	5,914,242.00
4_DH_3_S3	52,749,384.00	3,444,340.00	2,739,378.00
4_DS_1_S4	52,183,116.00	7,318,656.00	6,616,086.00
4_DS_2_S5	43,712,404.00	4,906,552.00	4,527,294.00
4_DS_3_S6	48,815,458.00	4,180,178.00	3,839,134.00
4_IgG_S10	30,956,338.00	776,590.00	594,992.00
4_K2_1_S17	45,992,640.00	15,045,056.00	14,129,568.00
4_K2_2_S18	27,370,070.00	9,026,356.00	8,379,914.00
4_K2_3_S19	52,109,530.00	21,389,512.00	20,195,410.00
4_K3_1_S20	29,391,002.00	2,025,650.00	1,697,494.00
4_K3_2_S21	43,535,218.00	3,826,188.00	3,223,030.00
4_K3_3_S22	48,075,330.00	3,967,366.00	3,283,742.00
4_WD_1_S7	80,362,542.00	25,203,878.00	18,439,628.00
4_WD_2_S8	98,528,138.00	25,490,912.00	19,413,628.00
4_WD_3_S9	53,818,474.00	2,500,754.00	2,011,166.00
8_K2_1_S23	39,711,548.00	21,397,248.00	18,582,456.00
8_K2_2_S24	40,101,454.00	26,623,774.00	24,153,352.00
8_K2_3_S25	32,906,498.00	20,369,976.00	18,568,684.00
8_K3_1_S26	38,113,642.00	9,817,790.00	7,920,204.00
8_K3_2_S27	38,152,118.00	11,029,236.00	8,797,988.00
8_K3_3_S28	46,712,314.00	25,661,040.00	20,207,194.00
12_K2_1_S29	36,976,508.00	13,069,650.00	12,505,044.00
12_K2_2_S30	43,545,256.00	14,900,536.00	14,182,790.00
12_K2_3_S31	38,718,196.00	15,670,436.00	14,791,794.00
12_K3_1_S32	43,479,688.00	4,752,668.00	3,837,618.00
12_K3_2_S33	31,205,306.00	1,026,976.00	885,716.00
12_K3_3_S34	25,404,798.00	1,837,676.00	1,448,170.00
16_K2_1_S35	56,577,908.00	16,183,532.00	15,469,104.00
16_K2_2_S36	38,347,870.00	12,287,338.00	11,730,840.00
16_K2_3_S37	30,119,956.00	11,670,598.00	11,138,836.00
16_K3_1_S38	31,721,220.00	2,218,408.00	1,833,964.00
16_K3_2_S39	27,345,420.00	994,878.00	859,788.00
16_K3_3_S40	31,164,748.00	646,304.00	520,608.00
20_K2_1_S41	37,693,126.00	10,777,708.00	10,177,506.00
20_K2_2_S42	41,456,140.00	13,934,760.00	12,857,290.00
20_K2_3_S43	30,775,094.00	8,892,270.00	8,256,114.00
20_K3_1_S44	29,213,312.00	8,071,824.00	7,547,998.00
20_K3_2_S45	47,298,070.00	12,319,872.00	11,120,578.00
20_K3_3_S46	63,796,638.00	19,151,266.00	17,635,486.00
V_DH_1_S47	38,354,362.00	35,656,150.00	28,585,514.00
V_DH_2_S48	41,777,574.00	36,364,144.00	30,996,082.00
V_DH_3_S49	37,077,836.00	28,418,822.00	24,390,660.00
V_DS_1_S50	48,050,330.00	45,020,544.00	43,058,582.00
V_DS_2_S51	41,674,098.00	38,302,310.00	36,794,930.00
V_DS_3_S52	43,751,872.00	39,941,036.00	38,095,330.00
V_IgG_S56	31,749,078.00	7,375,664.00	5,897,488.00

V_WD_1_S53	21,798,158.00	8,825,170.00	7,582,322.00
V_WD_2_S54	19,607,102.00	6,052,022.00	4,929,324.00
V_WD_3_S55	32,500,474.00	11,400,794.00	9,744,182.00
Z_DH_1_S57	61,270,690.00	16,125,394.00	12,245,922.00
Z_DH_2_S58	64,077,002.00	18,341,110.00	14,615,612.00
Z_DH_3_S59	67,469,872.00	29,555,502.00	23,851,138.00
Z_DS_1_S60	60,843,838.00	51,695,172.00	49,020,160.00
Z_DS_2_S61	53,028,422.00	32,888,186.00	29,970,752.00
Z_DS_3_S62	53,540,328.00	41,497,946.00	37,406,712.00
Z_IgG_S66	52,145,422.00	15,006,002.00	11,835,444.00
Z_WD_1_S63	51,181,810.00	34,580,236.00	27,206,620.00
Z_WD_2_S64	59,028,068.00	33,287,442.00	26,431,774.00
Z_WD_3_S65	60,486,502.00	20,390,910.00	16,681,584.00

Antibodies

H3K4me2: AM 39141, H3K4me3:Epicypher 13-0041, H3K4me3Q5his: Millipore ABE2570, WDR5: CST D9E1I, H3K4me3Q5ser: Millipore ABE2580, Rabbit IgG: Abcam ab172730, H3Q5his: Millipore ABE2578
All used 1:50 in antibody buffer

Peak calling parameters

MACS2 was used for peak calling with the following parameters (except for WDR5, which did not use --broad mode): macs2 callpeak \
-t ZT#AntibodyX.merged.bam \
-c ZT#IgG.bam \
-n ZT#AntibodyY_IgG_broad \
-f BAMPE -g mm (or hh) --keep-dup all \
--broad \
-q 0.05 \
--broad-cutoff .05 \
--outdir out_directory

Data quality

MACS2 peak calling was used with the corresponding IgG control. Peaks were visually inspected in IGV and by heatmap. Peak calling parameters were identical except in some cases where q values of .01 were used to reduce background peaks. Number of called peaks with q-values <.05 or .01 were used and are as follows (Peak-calling .broadPeak and .narrowPeak are available in the GEO submission):
singleHisHela_.BROAD.05_peaks.broadPeak: 17,981 Peaks
DualHisHela_.BROAD.05_peaks.broadPeak: 20,181 Peaks
K4me2_ZT0_IgG.BROAD.01_peaks.broadPeak: 58,790 Peaks
K4me3_ZT0_IgG.BROAD.05_peaks.broadPeak: 18,362 Peaks
WDR5_16_IgG.05_peaks.narrowPeak: 4,873 Peaks

Software

Sequencing files were demultiplexed using bcl2fastq2 (Illumina, v2.20). Samples were aligned to the hg19 or mm10 genome using bowtie2 (2.5.0), with the following parameters: --local --very-sensitive-local --phred33 -I 10 -X 700 --dovetail --no-unal --no-mixed --no-discordant. Low quality reads were filtered out using Samtools (1.9) with a cutoff MAPQ score of 30, and only unique reads kept for further processing. Unique read files for each replicate/timepoint/antibody were merged and used for peak calling using MACS2 (3.0.0a6) using the callpeaks function with the options -f BAMPE -q 0.05 --broad --broad-cutoff .05, using the corresponding IgG sample as the -c. For visualization, each sample was normalized by scaling the samples based off of the E. coli spike in, and genome coverage tracks (bigwig files) were produced using deepTools (3.5.1) bamCoverage function with the options –binSize 10 –smoothLength 30 --normalizeUsing None --scaleFactor # and using an ENCODE hg19 or mm10 blacklist file (https://doi.org/10.1038/s41598-019-45839-z) to discard regions with consistent non-specific signal. Additional code details can be found in the supplemental information
